# Education robot object detection with a brain-inspired approach integrating Faster R-CNN, YOLOv3, and semi-supervised learning

**DOI:** 10.3389/fnbot.2023.1338104

**Published:** 2024-01-04

**Authors:** Qing Hong, Hao Dong, Wei Deng, Yihan Ping

**Affiliations:** ^1^School of Mechanical Engineering, Nanjing Vocational University of Industry Technology, Nanjing, Jiangsu, China; ^2^Department of Economic Management, Shandong Vocational College of Science and Technology, Weifang, Shandong, China; ^3^Institute of Information Technology, Hunan Biological and Electromechanical Polytechnic, Changsha, Hunan, China; ^4^College of Computer Science, Northwestern University, Evanston, IL, United States

**Keywords:** educational robots, object detection, faster R-CNN, YOLOv3, semi-supervised learning, optimization

## Abstract

The development of education robots has brought tremendous potential and opportunities to the field of education. These intelligent machines can interact with students in classrooms and learning environments, providing personalized educational support. To enable education robots to fulfill their roles, they require accurate object detection capabilities to perceive and understand the surrounding environment of students, identify targets, and interact with them. Object detection in complex environments remains challenging, as classrooms or learning scenarios involve various objects, backgrounds, and lighting conditions. Improving the accuracy and efficiency of object detection is crucial for the development of education robots. This paper introduces the progress of an education robot's object detection based on a brain-inspired heuristic method, which integrates Faster R-CNN, YOLOv3, and semi-supervised learning. By combining the strengths of these three techniques, we can improve the accuracy and efficiency of object detection in education robot systems. In this work, we integrate two popular object detection algorithms: Faster R-CNN and YOLOv3. We conduct a series of experiments on the task of education robot object detection. The experimental results demonstrate that our proposed optimization algorithm significantly outperforms individual algorithms in terms of accuracy and real-time performance. Moreover, through semi-supervised learning, we achieve better performance with fewer labeled samples. This will provide education robots with more accurate perception capabilities, enabling better interaction with students and delivering personalized educational experiences. It will drive the development of the field of education robots, offering innovative and personalized solutions for education.

## 1 Introduction

In recent years, educational robots have been rapidly developing and being applied as innovative educational tools. Object detection is a crucial technology for educational robots as it helps them understand and perceive the surrounding environment, enabling better interaction with students and providing personalized learning experiences. Due to the complexity and diversity of educational settings, traditional object detection methods face several challenges in practical applications (Mittal et al., 2020). Existing object detection algorithms may encounter issues of false positives and false negatives. False positives refer to incorrectly recognizing non-target objects as target objects, while false negatives occur when actual target objects are not detected. These errors can have a negative impact on the perception capabilities and interaction effectiveness of educational robots (Mahajan et al., 2023). For example, if an educational robot mistakenly identifies a student's book as a cup, it may provide incorrect advice or guidance, thereby reducing the effectiveness of education. In an educational environment, the behavior and perspectives of students and teachers can change rapidly. Educational robots need to be able to quickly and accurately detect and recognize objects in real-time to provide timely feedback and interaction (Perez-Rua et al., 2020). Existing object detection algorithms often involve a trade-off between speed and accuracy. Some high-precision algorithms may require more computational resources and time (Zhang M. et al., 2022), making real-time performance challenging to achieve. On the other hand, faster algorithms may sacrifice accuracy, leading to less precise detection results. This poses a challenge for educational robots in choosing object detection algorithms as they need to strike a balance between speed and accuracy.

In the field of educational robot object detection, here are five common models:

1. Mask Region-based Convolutional Neural Networks [Mask R-CNN (Danielczuk et al., 2019)]: Mask R-CNN not only performs object detection and localization but also generates semantic segmentation masks for each detected object. It adds a segmentation branch to the base object detection model to predict pixel-level masks. It is slower compared to models that only perform object detection.

2. Fully Convolutional One-Stage Object Detection [FCOS Tian et al., 2022] is a fully convolutional one-stage object detection algorithm that achieves better object detection performance by performing dense predictions on feature maps.

3. Single Shot MultiBox Detector (SSD): SSD is a single-shot object detection model that predicts object locations and classes on different feature maps with varying scales. It has a high detection speed and good accuracy but may encounter difficulties in handling small objects. The drawbacks of FCOS include high computational cost, poor scale invariance, inaccurate localization, difficulty in handling dense objects, and sensitivity to variations in aspect ratios.

4. RetinaNet (Afif et al., 2020): RetinaNet is an object detection model based on the Feature Pyramid Network (FPN), which enables detection of objects at different scales using a multi-level feature pyramid. It performs well in detecting small and densely packed objects but is slower in speed.

5. EfficientNet (Atila et al., 2021): EfficientNet is an efficient and accurate convolutional neural network architecture that exhibits high accuracy in the field of object detection while having low computational costs and moderate parameter count. Its drawback is that it is relatively complex, which may increase the complexity of model training and deployment.

We aim to find a method to improve the efficiency of object detection in education robots to meet real-time requirements. To overcome these challenges, we propose integrating Faster R-CNN, YOLOv3, and semi-supervised learning methods and optimizing them (Sun, 2022). We construct a diverse dataset and annotate bounding boxes for the objects of interest. This dataset will be used for training and evaluating our proposed object detection algorithm. Next, we delve into the architecture and implementation details of Faster R-CNN and YOLOv3, comparing their advantages and disadvantages, and integrate these two algorithms to leverage their strengths and enhance object detection capabilities. To further improve model performance, we introduce semi-supervised learning techniques (He and Tang, 2023). We utilize unlabeled data and employ self-training, co-training, and pseudo-labeling methods to augment the training process of the model (Liu Y.-C. et al., 2022). Additionally, we apply transfer learning by initializing the model with pre-trained weights to enhance its robustness (Liu C. et al., 2022). Data augmentation techniques such as random cropping, flipping, and rotation are also employed to increase the diversity and quantity of the data (Wang et al., 2023). By evaluating the optimized models on an independent validation dataset, we validate the effectiveness of this approach (Sundermeyer et al., 2020). Experimental results demonstrate significant improvements in the performance, accuracy, and robustness of our object detection algorithm for education robots in complex environments. This will provide education robots with more accurate perception capabilities, enabling better interaction with students and delivering personalized educational experiences. It will drive the development of the field of education robots, offering innovative and personalized solutions for education.

The contribution points of this paper are as follows:

- We have designed an integrated framework that combines two object detection algorithms, Faster R-CNN and YOLOv3, to complement each other. Faster R-CNN excels in object localization and classification accuracy, while YOLOv3 offers higher detection speed and adaptability. By integrating these two algorithms, our system achieves a balance between accuracy and efficiency, enhancing the performance of object detection.
- To address the difficulties in data collection and annotation for object detection tasks in educational robot applications, we have introduced a semi-supervised learning approach. This approach leverages information from unlabeled data to enhance the generalization ability of the object detection model, further improving accuracy. By making full use of limited labeled data and abundant unlabeled data, the system can better adapt to changes in the educational environment, enhancing the performance of object detection.
- We have explored the potential application of brain-inspired methods in educational robot object detection systems. By simulating the processing mechanisms and structures of the human brain, the system can better understand and recognize targets in the educational environment, providing strong support for intelligent-assisted teaching. This approach has shown significant improvements in experiments, demonstrating its effectiveness and feasibility, and providing valuable exploration and practice for the intelligence and automation of educational robots.

In the second section, we presented related work, described our proposed research methodology, and conducted discussions. The third section introduced the main methods of this paper, such as the Faster R-CNN, YOLOv3, and Semi-supervised Learning. In the fourth section, we discussed the experimental part, including comparisons, ablation experiments, and visualizations. The fifth section presented the discussion, elaborating on the methodology and recent advancements in the field, highlighting the limitations of our approach, and providing insights into future work. Finally, in the sixth section, we summarized the methodology and provided a conclusive summary.

## 2 Related work

Education robot object detection refers to the use of machine learning and computer vision techniques in the field of education to identify and locate specific objects in images or videos (Alam, 2022). Through object detection, education robots can automatically recognize and analyze various objects in educational settings, such as books, stationery, and laboratory equipment, thereby providing more intelligent teaching support and interactive experiences. The emergence and development of education robot object detection can be traced back to related research in the fields of computer vision and artificial intelligence. Computer vision is the discipline that studies how computers can acquire, process, and understand visual information, and object detection is an important task within this field.

Early object detection methods primarily relied on manually designed feature extraction and classifiers. For example, Haar features and cascade classifiers (Xu X. et al., 2020) were commonly used methods that had efficient detection speed and were suitable for real-time applications in robot object detection. Cascade classifiers reduce computation by decomposing complex tasks and quickly filtering out non-target regions. They perform well in detecting small objects and have lower demands in resource-limited environments. However, compared to deep learning methods (Chen F. et al., 2019). Haar features and cascade classifiers have lower detection accuracy because Haar features cannot effectively capture the complex textures and shapes of targets. They are sensitive to target poses and lighting variations, which may lead to a decline in detection performance. Training cascade classifiers requires a large amount of annotated data, posing challenges in certain specific domain object detection tasks where complex scenes and diverse targets are present.

With the rise of deep learning technology, significant breakthroughs have been made in object detection. Deep learning utilizes multi-layer neural networks for feature learning and pattern recognition, enabling automatic feature representation learning from data, thereby improving the accuracy and robustness of object detection. Among them, Convolutional Neural Networks (CNNs) (Kattenborn et al., 2021) serve as a core technology in deep learning and provide powerful tools for object detection. CNNs were initially proposed in the late 1980s and early 1990s. Yann LeCun and others introduced a CNN architecture called LeNet-5 (Islam and Matin, 2020) in 1989 for handwritten digit recognition tasks. LeNet-5 is a classic CNN model that includes basic components such as convolutional layers (Luo Z. et al., 2019), pooling layers, and fully connected layers, laying the foundation for the subsequent development of deep learning. In object detection tasks, CNNs are typically used as feature extractors. By training on large-scale datasets, CNNs can learn rich and discriminative feature representations. These learned features can then be fed into subsequent classifiers or regressors to accomplish the object detection task. With the advancement of technology and increased computational power, CNNs have achieved great success in the field of object detection. Particularly in 2012, the AlexNet (Ismail Fawaz et al., 2020) model made a breakthrough in the ImageNet image classification challenge, leading to a renaissance of deep learning in the field of computer vision. Since then, many CNN-based object detection algorithms have been proposed, such as R-CNN (Bharati and Pramanik, 2020), Fast R-CNN (Maity et al., 2021), and YOLO (Jiang et al., 2022). Region-based Convolutional Neural Networks (R-CNN) is a region-based object detection algorithm that uses methods like selective search to extract candidate regions, performs feature extraction and classification for each candidate region, and then uses a regressor for precise localization of objects. While it excels in accuracy, it is relatively slow in speed. Fast R-CNN is an improvement over R-CNN, achieving end-to-end training by introducing Region of Interest (RoI) pooling layers. It takes the entire image as input, shares convolutional features to extract features for candidate regions, and uses classifiers and regressors for object recognition and localization. Compared to R-CNN, Fast R-CNN offers faster detection speed and improved accuracy. You Only Look Once (YOLO) is a one-stage detection-based object detection algorithm. It formulates the object detection task as a regression problem, directly predicting the class and bounding box of objects on the image. YOLO has fast detection speed and can be applied in real-time scenarios such as video streams, but it suffers from some accuracy loss in detecting small and densely packed objects. With further developments in deep learning, models such as CenterNet (Xu Z. et al., 2020), EfficientDet (Mekhalfi et al., 2021), and EfficientPose (Groos et al., 2021) have been proposed for object detection or human pose estimation tasks using deep neural networks. CenterNet, introduced by the research team at the University of California, Berkeley in 2019, is a center-point-based object detection algorithm that performs object detection by predicting the center point positions and bounding box sizes of objects. In the field of education robots, CenterNet can be used to detect students' keypoints or regions of interest, such as facial expressions and eye gaze points, to help the robot perceive the students' states and needs. It has advantages such as efficiency, accuracy, multitasking, and adaptability to small targets. However, it faces challenges in handling dense targets, occlusions, and pose variations, and requires a large amount of annotated data for training. EfficientDet, introduced by the Google Brain team in 2020, builds an efficient and accurate object detection framework based on the EfficientNet architecture using Bi-directional Feature Pyramid Network (BiFPN) to construct a feature pyramid network with multiple scales. In the field of education robots, EfficientDet can be used to identify and locate objects or learning tools around the robot, enabling interaction and teaching with students. It is an efficient and accurate object detection model suitable for object recognition and localization in educational robots. However, it also requires a large amount of annotated data and has limitations in handling dense targets. EfficientPose, proposed by the research team at the Chinese University of Hong Kong in 2021, is an algorithm for human pose estimation. It utilizes EfficientNet and a top-down branch to detect and estimate the positions of human keypoints to infer human poses. In the field of education robots, EfficientPose can be used to recognize students' postures and actions, such as correct sitting posture and raising hands, providing real-time feedback and guidance. It is an efficient and accurate pose and action recognition model that can be used for real-time feedback and guidance on student postures. It also requires a large amount of annotated data and has limitations in complex poses and actions. However, deep learning-based object detection methods also have limitations. These methods often require a large amount of annotated data for training, including annotations for object categories and bounding boxes, which can be time-consuming and resource-intensive in the field of education robots. The performance of deep learning models heavily relies on parameter settings and tuning, requiring iterative experiments and optimization to achieve good detection results. Deep learning models typically require training and inference on high-performance computing devices, posing challenges for education robot systems with limited resources.

Weakly supervised learning methods (Li et al., 2019) have provided effective solutions for object detection tasks in the field of education robots, significantly reducing the cost and workload of data annotation. Traditional object detection methods typically require a large amount of detailed annotated data, including manually labeling the position and class information of each object instance. Acquiring a large-scale annotated dataset can be challenging, making weakly supervised learning methods a feasible alternative. Weakly supervised learning methods train object detection models by utilizing coarse labels or auxiliary information, thereby reducing the need for detailed annotation data. In the field of education robots, weakly supervised learning methods can be applied in several aspects. Firstly, weak labels can be used. Weak labels refer to labels that provide only object category information without detailed bounding box information. By collecting images with object category labels in educational robot scenarios, weakly supervised learning methods can be used to train object detection models. These methods significantly reduce the annotation workload and improve training efficiency. Another weakly supervised learning method is Multiple Instance Learning (MIL) (Zhang H. et al., 2022), which uses a set of instances to represent the presence of an object. In education robots, MIL methods can be used for object detection. By collecting a set of images containing the target object and a set of images without the target object, MIL methods can be used to train object detection models. This enables the model to learn the features and contextual information of the object from the examples, thereby improving the accuracy of object detection. Weakly supervised learning methods have limitations in terms of accuracy and robustness. Compared to methods trained with detailed annotation data, weakly supervised learning methods generally perform worse in object detection accuracy and robustness. This is because the label information used in weakly supervised learning methods is relatively coarse and cannot provide precise object positions and bounding boxes. In applications that require high precision, weakly supervised learning methods may not achieve the desired results (Zheng et al., 2022). Customizing weak supervision strategies for different educational robot tasks and scenarios is necessary to fully leverage the auxiliary information during the training process. This requires further research and experimental validation to find the most suitable weak supervision strategies for specific tasks. To provide a more comprehensive and accurate understanding of objects, enhance the teaching effectiveness and learning experience of education robots, researchers have proposed cross-modal object detection methods (Li et al., 2020). Cross-modal object detection methods enable the fusion of multimodal data. Education robots often utilize various perception modalities such as images, speech, and text. By integrating data from these different modalities, richer information can be obtained to understand students' behaviors and needs. By simultaneously analyzing students' speech commands and image inputs, education robots can better comprehend students' intentions and provide appropriate teaching feedback. Cross-modal object detection methods can facilitate cross-modal contextual understanding. This approach not only detects and recognizes objects but also leverages the correlations between different modalities to understand the contextual information of objects. By analyzing the surrounding environmental images and speech interactions of students, education robots can better understand the learning context and provide personalized teaching guidance based on the context. Cross-modal object detection methods can also make use of cross-modal attention mechanisms. These mechanisms can automatically learn and adjust the importance of different modalities, thereby better focusing on information relevant to the target objects. In education robots, cross-modal attention mechanisms can be used to concentrate attention on teaching materials or student behaviors related to the learning tasks, thereby improving teaching effectiveness and learning efficiency. Cross-modal object detection methods require solutions for handling heterogeneity and incompleteness between different modalities in data fusion. Acquiring and annotating large-scale cross-modal datasets is a challenging task, and the data volume from different modalities may be imbalanced.

Due to the diversity and complexity of objects in educational environments, including differences in shape, color, and size, algorithms need to have the ability to recognize and locate various types of objects. The presence of complex backgrounds and occlusions also adds difficulty to object detection, as algorithms must distinguish objects from the background and accurately locate them in the presence of occlusions. Education robots require efficient processing of object detection tasks to meet real-time or near-real-time teaching demands. Overcoming these challenges requires algorithms with strong generalization capabilities, object segmentation and boundary detection abilities, efficient processing speeds, and the ability to make effective use of limited datasets and accurate annotation data. Therefore, leveraging the advantages of deep learning and improving object detection challenges specific to education robots is an important research direction. By conducting in-depth research and applying brain-inspired methods to object detection, it is expected to contribute to both research and practical applications in this field.

Brain-inspired methods (Zendrikov et al., 2023) refer to approaches that draw inspiration from the structure and functionality of the human brain and apply these principles and mechanisms to the fields of computer science and artificial intelligence. Education robots are robots that utilize artificial intelligence technologies to assist in the education and learning process. Object detection is an essential task in the field of computer vision, aiming to accurately identify and locate specific objects in images or videos. When applying brain-inspired methods to object detection tasks in education robots, we can explore and leverage the structure and functionality of the human brain from multiple perspectives to improve the performance and efficiency of object detection. Designing brain-inspired neural network architectures is a crucial aspect. We can draw insights from the visual processing mechanisms in the human brain and create neural network models with similar structures and connectivity patterns to simulate the transmission and processing of visual information. For example, we can design hierarchical neural networks where each layer corresponds to different visual processing stages in the human brain. Such models can perform object detection tasks by progressively extracting features and performing object classification. Short-term memory and context modeling are also key aspects of brain-inspired methods in object detection for education robots. The human brain possesses short-term memory capabilities, allowing it to remember and utilize observed objects or contextual information to better understand the current scene. Education robots can leverage this mechanism by utilizing previously observed objects or contextual information in object detection tasks, thereby improving the understanding of objects in the current scene and enhancing the accuracy of object detection. Brain-inspired methods can also improve object detection performance through the fusion of heterogeneous information. Education robots can utilize multiple perceptual modalities (such as vision and audition) and multiple sources of information for fusion to obtain a more comprehensive understanding of objects. Brain-inspired methods can draw inspiration from the collaborative work of multiple brain regions in the human brain, integrating and analyzing data from different perceptual modalities and information sources to improve the robustness and accuracy of object detection. By combining brain-inspired methods with education robots, we can achieve more intelligent and efficient object detection systems, bringing innovation and improvements to the field of education.

## 3 Methodology

### 3.1 Overview of our network

The objective of this work is to optimize and implement an object detection algorithm for education robots by integrating Faster R-CNN, YOLOv3, and semi-supervised learning (Chen J. et al., 2019). The goal is to improve the accuracy and efficiency of object detection in complex environments, enabling education robots to perceive and understand their surroundings, identify targets, and interact with them effectively.

**Figure 1:**
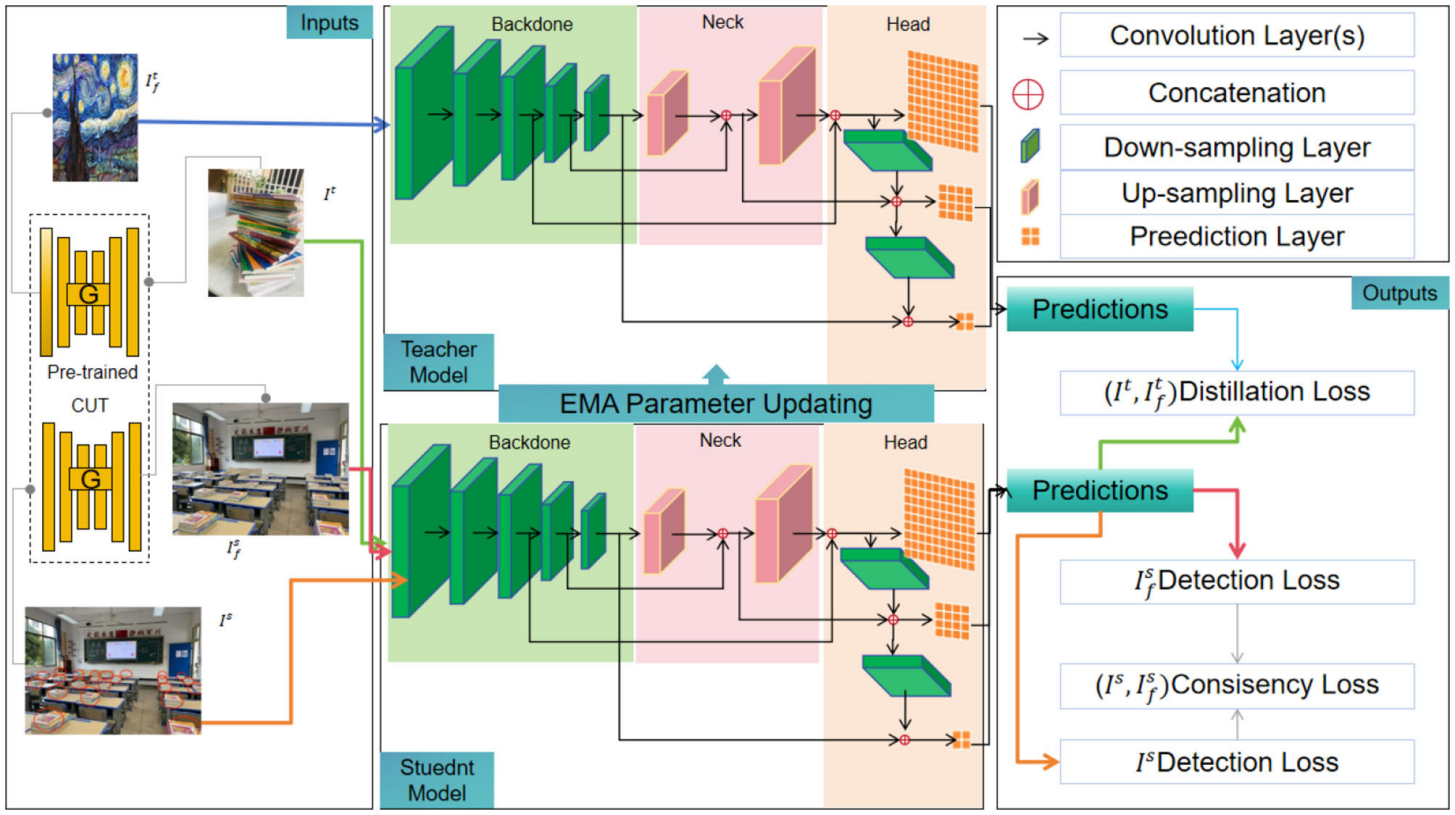
The overall framework diagram of the proposed mode.

Figure 1 shows the overall framework diagram of the proposed model.

Overall workflow of the method:

1. Data collection and preprocessing: collect multimodal data, including images and speech data, that involve students and educational scenarios. Preprocess the image data, including resizing the images to a fixed size, performing color space conversion (e.g., RGB to grayscale or HSV), and applying data augmentation techniques such as random cropping, flipping, rotation, etc., to increase data diversity and model robustness.

2. Feature extraction and representation learning: use pretrained Faster R-CNN and YOLOv3 models as the base networks to extract region features and detection box information from the images. For Faster R-CNN, obtain candidate regions and their corresponding feature vectors by running the base network and the Region Proposal Network on the images. For YOLOv3, input the images into the network, extract feature maps at different scales through convolutional and pooling layers, and extract the target's feature representation from the feature maps. Perform representation learning on the extracted feature vectors, which can involve dimensionality reduction techniques such as principal component analysis (PCA) or other feature selection and extraction methods to obtain more discriminative representations.

3. Brain-inspired candidate object box generation: generate candidate object detection boxes using brain-inspired methods. This approach can simulate human perception and cognition processes by incorporating visual features, contextual information, and prior knowledge to generate candidate boxes with potential targets. Heuristic rules or algorithms can be employed to determine the positions and sizes of the candidate boxes, such as edge detection, color segmentation, sliding windows, etc.

4. Training of semi-supervised object detection model: train the object detection model using semi-supervised learning techniques. Perform supervised training using labeled data. Input the labeled data into the object detection model, perform object classification and position adjustment through the loss function, and optimize the model parameters. Then, perform unsupervised training using unlabeled data. Self-training, co-training, or other methods can be employed to iteratively train the model using unlabeled data and the model's output, further enhancing the model's performance.

5. Object detection and result output: during the testing phase, apply the trained object detection model to new image data. Perform object classification and position adjustment on the candidate boxes in the image to achieve object detection and localization. Output the detection results, which can include labels of the object categories and positions, or visualized bounding boxes indicating the object locations in the image, for application and interaction in educational robots.

By integrating the feature extraction capabilities of Faster R-CNN and YOLOv3, combining the training method of semi-supervised learning, and incorporating brain-inspired methods for generating candidate object detection boxes, this method can improve the accuracy, robustness, and generalization ability of object detection in educational robots (Zhou et al., 2023). Semi-supervised learning can reduce the need for a large number of labeled samples, enhancing the scalability and universality of the model.

### 3.2 Faster R-CNN

Faster Region-based Convolutional Neural Network (R-CNN) is a popular object detection algorithm known for its high accuracy. It consists of two main components: a region proposal network (RPN) and a detection network.

**Figure 2:**
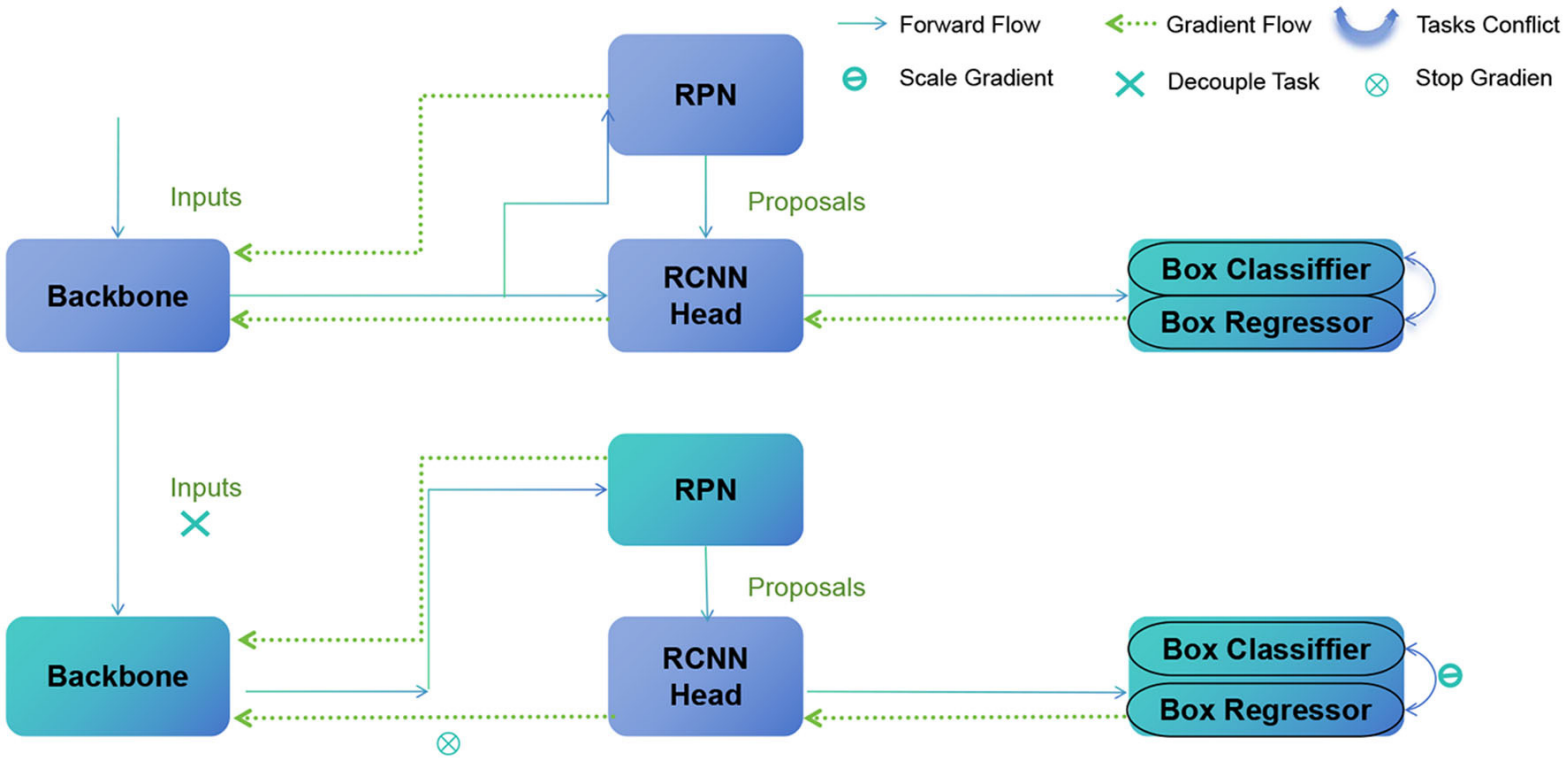
A schematic diagram of the Faster R-CNN.

Figure 2 shows the overall framework diagram of the proposed model.

The basic principle of Faster R-CNN involves the following steps:

- Base network: use a pre-trained convolutional neural network to extract features from the input image.
- Region proposal network (RPN): the RPN generates candidate target regions by sliding windows on the feature map, treating them as anchor boxes, and predicting whether the anchor boxes contain objects and how to adjust the boundaries of the anchor boxes.
- Region of interest pooling (RoI Pooling): divide each candidate region into fixed-size sub-regions and map them onto a fixed-size feature map.
- Object classification network: use a fully connected network to classify each candidate region, taking the output of RoI Pooling as input, and output the probabilities of each candidate region belonging to different target classes.
- Bounding box regression: perform bounding box regression to adjust the coordinates of the target's bounding box in each candidate region.

In order to perform object detection in Faster R-CNN, we employ the Region Proposal Network (RPN) for generating candidate regions. The RPN stage involves two key formulas, one for calculating the coordinates of anchor boxes and another for computing the loss between anchor boxes and the ground truth bounding boxes.

Firstly, we use the following formulas to calculate the coordinates of anchor boxes and determine their positions in the image:


(1) $$\begin{array}{l} \begin{array}{c} x_{\text{anchor}}=x_{\text{center}}-\frac{w_{\text{anchor}}}{2}\\ y_{\text{anchor}}=y_{\text{center}}-\frac{h_{\text{anchor}}}{2}\\ w_{\text{anchor}}={\text{width}}_{\text{anchor}}\\ h_{\text{anchor}}={\text{height}}_{\text{anchor}} \end{array} \end{array}$$

*x*_anchor_ and *y*_anchor_: The top-left coordinates of the anchor box, indicating its position in the image. *x*_center_ and *y*_center_: The coordinates of the center point of the target or anchor box, used to determine the position of the anchor box. *w*_anchor_ and *h*_anchor_: The width and height of the anchor box, used to determine its size. width_anchor_ and height_anchor_: The predefined width and height of the anchor box, typically set as fixed values during training. Building upon this, we introduce the Smooth L1 Loss as the loss function between anchor boxes and the ground truth bounding boxes. The computation formula for this loss function is as follows:


(2) $$\begin{array}{l} L_{\text{bbox}}=\underset{i}{\sum }L_{\text{smooth}}\left(t_{i}-t_{i}^{\prime },{\text{1}}_{i\text{ is positive}}\right) \end{array}$$

where *t*_*i*_ represents the predicted bounding box offset, $t_{i}^{\prime }$ represents the corresponding ground truth bounding box offset, and **1**_*i*is positive_ is an indicator function that takes a value of 1 when the anchor box *i* is a positive sample. In Faster R-CNN, there is also a crucial step of calculating the output of the RoI (Region of Interest) pooling layer. The RoI pooling layer is used to map RoIs of different sizes onto a fixed-size feature map to maintain spatial alignment of RoI features. We use the following formula to compute the output of the RoI pooling layer:


(3) $$\begin{array}{l} F_{\text{roi}}=\text{RoI pooling}\left(F_{\text{conv}},\text{p}\right) \end{array}$$

where *F*_conv_ represents the convolutional feature map obtained from the feature extraction network, and **p** represents the input parameters for the RoI pooling layer, including the coordinates and size information of the RoI. The RoI pooling layer maps RoIs of different sizes onto a fixed-size feature map to maintain spatial alignment of RoI features.

The purpose of this step is to perform pooling operations on the feature map regions corresponding to RoIs of different sizes, resulting in fixed-size RoI features. This allows mapping RoIs of different sizes onto the same-sized feature map, facilitating subsequent object classification and bounding box regression. Faster R-CNN also includes a detection network for object classification and bounding box regression. The detection network takes the candidate boxes from the Region Proposal Network (RPN) as input and performs object classification and bounding box regression on them. We use the following formulas to compute the outputs of the detection network. The formula for object classification is as follows:


(4) $$\begin{array}{l} F_{\text{cls}}=\text{softmax}\left(W_{\text{cls}}\cdot F_{\text{roi}}+b_{\text{cls}}\right) \end{array}$$

The formula for bounding box regression is as follows:


(5) $$\begin{array}{l} F_{\text{reg}}=W_{\text{reg}}\cdot F_{\text{roi}}+b_{\text{reg}} \end{array}$$

where *W*_cls_, *b*_cls_, *W*_reg_, and *b*_reg_ are learned parameters. The softmax function is used to convert the object classification output into a probability distribution over classes.

Finally, we use the object classification results and bounding box regression results to filter out the final detection results. By setting a threshold, we select the target boxes with high confidence as the final detection results and refine their bounding box positions using the bounding box regression results for more accurate localization. By integrating the RPN and detection network, Faster R-CNN achieves precise object detection and has shown significant performance improvements on multiple benchmark datasets.

### 3.3 YOLOv3

You Only Look Once version 3 (YOLOv3) is another popular object detection algorithm known for its real-time processing speed. It divides the input image into a grid and predicts bounding boxes and class probabilities directly from the grid cells (Luo H.-W. et al., 2019). YOLOv3 improves upon its predecessor by introducing various architectural changes and feature extraction techniques.

**Figure 3:**
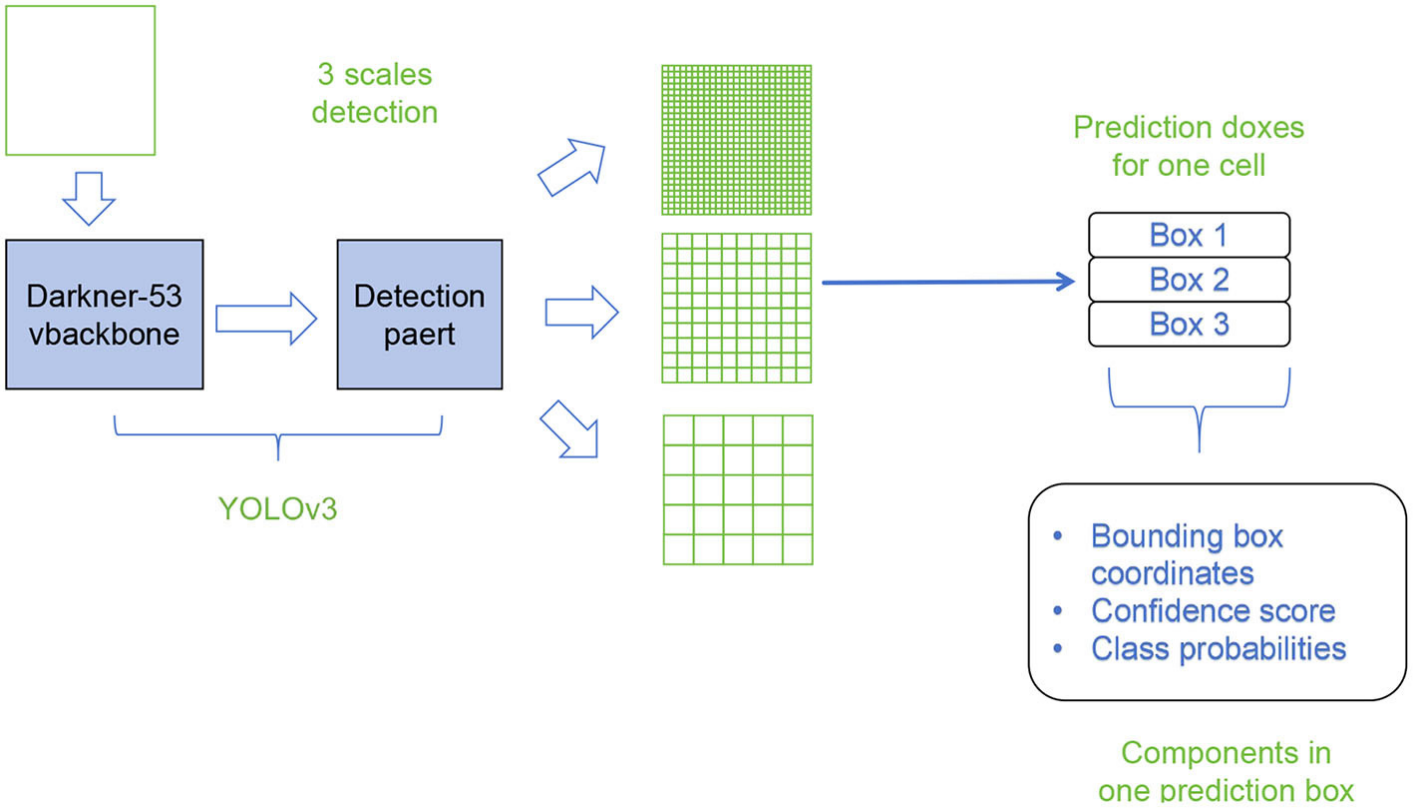
A schematic diagram of the YOLOv3.

Figure 3 shows the overall framework diagram of the proposed model.

The basic principle of YOLOv3 involves the following steps:

- Network architecture: a deep convolutional neural network based on Darknet is used, which includes multiple convolutional layers and fully connected layers to extract features from input images.
- Feature extraction: the input image is downsampled multiple times through convolutional layers to obtain feature maps at different scales, capturing the details and contextual information of the targets.
- Grid division: the input image is divided into a set of regular grid cells, and each cell is responsible for detecting the presence of objects in that region.
- Anchor box definition: multiple predefined anchor boxes with different sizes and aspect ratios are defined for each grid cell, capturing objects of different sizes and shapes.
- Object detection: for each anchor box, the network predicts the class probabilities and bounding box coordinates of the target through the output of the convolutional layers. This process is performed on feature maps at different scales, combining multi-scale predictions to obtain global object detection results.
- Non-maximum suppression (NMS): since the same object may be detected by multiple anchor boxes, non-maximum suppression algorithm is used to eliminate overlapping detection results. The most accurate bounding boxes are selected based on the class probabilities and overlap.

In the proposed method, YOLOv3 is integrated to leverage its real-time processing speed. By combining YOLOv3 with Faster R-CNN, the algorithm aims to achieve a balance between accuracy and real-time performance. YOLOv3's efficient grid-based approach allows for faster inference, making it suitable for real-time applications (Liu et al., 2021). It complements the accuracy of Faster R-CNN, enhancing the overall object detection capabilities of the education robot. The integrated model benefits from the speed advantages of YOLOv3 and the accuracy of Faster R-CNN, leading to improved real-time object detection in complex environments.

Anchor Box is an important concept in the YOLOv3 algorithm, used to predefine default bounding boxes. Each Anchor Box is represented by its width (*w*_*k*_) and height (*h*_*k*_) and is used to predict the position and size of the target. Feature maps are image representations generated by convolutional neural networks (CNNs) and are used in the YOLOv3 algorithm to detect objects and provide their position and feature information.

YOLOv3 uses the following equation to predict the position and confidence of the target bounding boxes:


(6) $$\begin{array}{l} {\text{B}}_{ijk}=\left(t_{x},t_{y},t_{w},t_{h},t_{o}\right) \end{array}$$

where *t*_*x*_ and *t*_*y*_ represent the offsets of the bounding box center relative to the feature map cell, *t*_*w*_ and *t*_*h*_ represent the width and height of the bounding box, and *t*_*o*_ represents the confidence of whether the bounding box contains the target. Class prediction is another important aspect of the YOLOv3 algorithm, which uses the following equation to predict the class probabilities of the targets:


(7) $$\begin{array}{l} {\text{P}}_{ij}=\left(p_{1},p_{2},...,p_{C}\right) \end{array}$$

where *p*_1_, *p*_2_, ..., *p*_*C*_ represent the probabilities of different classes. To train the YOLOv3 algorithm, a loss function needs to be defined to measure the difference between the predicted results and the ground truth labels. YOLOv3 uses localization loss and classification loss to optimize object detection performance. The formula for localization loss is as follows:


(8) $$\begin{array}{l} L_{\text{loc}}=\sum_{i=0}^{S^{2}}\sum_{j=0}^{B}{⊮}_{ij}^{\text{obj}}[{\left(t_{x}-{\hat{t}}_{x}\right)}^{2}+{\left(t_{y}-{\hat{t}}_{y}\right)}^{2}+{\left(t_{w}-{\hat{t}}_{w}\right)}^{2}\\ +{\left(t_{h}-{\hat{t}}_{h}\right)}^{2}] \end{array}$$

where ${⊮}_{ij}^{\text{obj}}$ indicates whether the bounding box *i, j* contains an object, $\left({\hat{t}}_{x},{\hat{t}}_{y},{\hat{t}}_{w},{\hat{t}}_{h}\right)$ represents the predicted bounding box, and (*t*_*x*_, *t*_*y*_, *t*_*w*_, *t*_*h*_) represents the ground truth bounding box. The formula for classification loss is as follows:


(9) $$\begin{array}{l} L_{\text{cls}}=\overset{S^{2}}{\underset{i=0}{\sum }}\overset{B}{\underset{j=0}{\sum }}{⊮}_{ij}^{\text{obj}}\overset{C}{\underset{c=1}{\sum }}{\left(p_{c}-{\hat{p}}_{c}\right)}^{2} \end{array}$$

where ${⊮}_{ij}^{\text{obj}}$ indicates whether the bounding box *i, j* contains an object, *p*_*c*_ represents the predicted class probability, and ${\hat{p}}_{c}$ represents the ground truth class probability.

### 3.4 Semi-supervised Learning

Semi-supervised learning is a machine learning approach that aims to leverage both labeled and unlabeled data to improve model performance (Park et al., 2022). In traditional supervised learning, models are trained solely on labeled data, which can be expensive and time-consuming to obtain. Semi-supervised learning addresses this limitation by incorporating unlabeled data during the training process (Scoullos et al., 2018).

**Figure 4:**
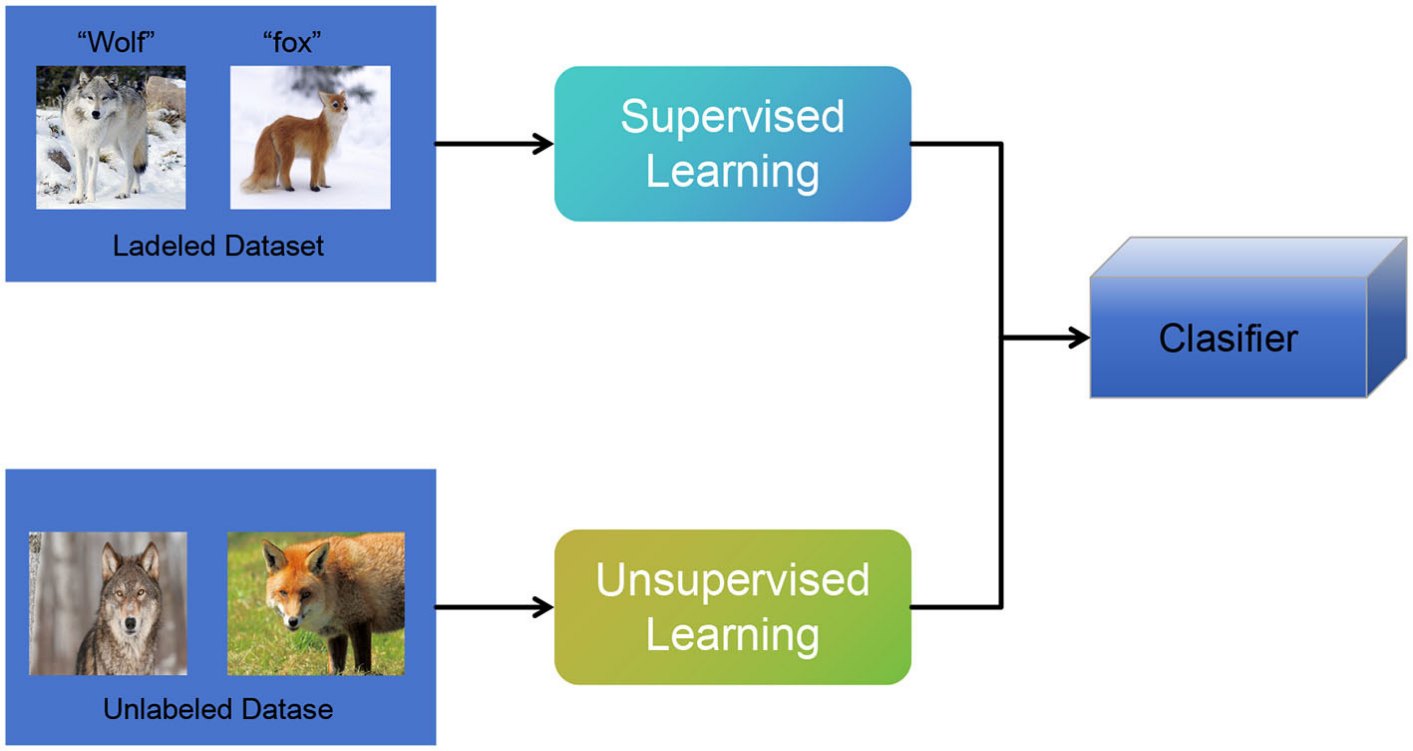
A schematic diagram of the Semi-supervised Learning.

Figure 4 shows the overall framework diagram of the proposed model.

The basic principle of Semi-supervised Learning involves the following steps:

- Network architecture: a deep convolutional neural network based on Darknet is utilized, comprising multiple convolutional layers and fully connected layers, to extract features from the input image.
- Feature extraction: the input image is downsampled multiple times through convolutional layers, generating feature maps at different scales to capture fine details and contextual information of the objects.
- Grid division: the input image is divided into a set of regular grid cells, where each cell is responsible for detecting objects within its assigned region.
- Anchor box definition: multiple predefined anchor boxes with different sizes and aspect ratios are defined for each grid cell, aiming to capture objects of various sizes and shapes.
- Object detection: for each anchor box, the network predicts the class probabilities and bounding box coordinates of the object using the output of the convolutional layers. This process is performed on feature maps at different scales, and the predictions from multiple scales are combined to obtain global object detection results.
- Non-maximum suppression (NMS): as multiple anchor boxes may detect the same object, non-maximum suppression algorithm is employed to eliminate overlapping detection results. the most accurate bounding boxes are selected based on the class probabilities and overlap scores.

In the proposed method, semi-supervised learning plays a crucial role in utilizing both labeled and unlabeled data to improve the model's performance (Tang, 2022). By training on a combination of labeled and unlabeled data, the model can learn more robust and generalizable representations. The unlabeled data provides additional information and helps the model capture the underlying structure of the data more effectively. This approach is particularly useful in scenarios where obtaining labeled data is challenging or expensive.

In the context of the specific application, the semi-supervised learning model aims to enhance the educational robot's capabilities by leveraging both labeled and unlabeled data (Ezeonu et al., 2023). By incorporating unlabeled data from educational scenarios, the model can learn to recognize and understand patterns, behaviors, and interactions that are specific to the educational context. This enables the model to provide more accurate and personalized educational support to students, based on the knowledge and insights gained from the unlabeled data.

The formula in Semi-supervised learning is as follows:


(10) $$\begin{array}{l} L\left(\theta \right)=L_{labeled}\left(\theta_{labeled}\right)+\lambda L_{unlabeled}\left(\theta_{unlabeled}\right) \end{array}$$

In Eq. 10, *L*(θ) represents the overall loss function of the semi-supervised learning approach. It consists of two terms: the labeled data loss *L*_*labeled*_(θ_*labeled*_) and the unlabeled data loss *L*_*unlabeled*_(θ_*unlabeled*_).

The variables and parameters in the equation are defined as follows:

- θ: the set of all trainable parameters in the model. - θ_*labeled*_: the subset of parameters used for labeled data. - θ_*unlabeled*_: the subset of parameters used for unlabeled data. - λ: a balancing parameter that controls the relative importance of the labeled and unlabeled data losses.

The labeled data loss *L*_*labeled*_(θ_*labeled*_) measures the discrepancy between the model's predicted outputs and the ground truth for labeled data. It is typically calculated using a loss function such as cross-entropy loss or mean squared error, depending on the task at hand.

The unlabeled data loss *L*_*unlabeled*_(θ_*unlabeled*_) leverages the unlabeled data to improve the model's performance. This loss term encourages the model to produce consistent predictions on similar unlabeled samples. The specific form of this loss depends on the semi-supervised learning algorithm used, such as the consistency loss or the entropy minimization loss.

The Semi-supervised Learning approach combines the labeled and unlabeled data losses to jointly optimize the parameters θ_*labeled*_ and θ_*unlabeled*_ using techniques like gradient descent or other optimization algorithms. By leveraging the large amounts of unlabeled data, this approach aims to enhance the model's performance and improve its generalization capabilities.

## 4 Experiment

The following experimental setup was used for conducting simulated experiments on object detection in this study: We created a virtual environment and scenes using the Unity engine. We utilized the CARLA simulator to configure various parameters and object properties in the virtual environment. The Raspberry Pi served as the control unit. We used TensorFlow Lite to load and run the object detection model.

### 4.1 Datasets

The data sets selected in this paper are: Common Objects in Context (COCO) dataset (Deng et al., 2023), Pascal VOC dataset (Liu et al., 2023), ISPRS test project Udacity AI for Robotics Dataset (Ribeiro et al., 2023), ImageNet Dataset (Liu et al., 2023).

Common Objects in Context (COCO) dataset:

The COCO dataset is a widely used dataset for tasks such as object detection, segmentation, and image captioning. It consists of over 330,000 images covering 80 common object categories, including people, cars, animals, furniture, and more. Each image is annotated with precise object bounding boxes and segmentation masks for object instances. Additionally, the COCO dataset provides annotations with five descriptive sentences for image captioning tasks. The COCO dataset is extensively used for training and evaluation in computer vision tasks such as object detection, image segmentation, and image generation.

Pascal VOC dataset:

The Pascal Visual Object Classes (VOC) dataset is a classic dataset for object detection and semantic segmentation tasks. It includes 20 common object categories, such as people, cars, airplanes, animals, and more. The Pascal VOC dataset provides ~10,000 annotated images for object detection and semantic segmentation tasks. Each image is annotated with precise object bounding boxes and segmentation masks for object instances. The Pascal VOC dataset is widely used in computer vision research, especially for the development and evaluation of object detection and segmentation algorithms.

Udacity AI for Robotics dataset:

The Udacity AI for Robotics dataset is designed specifically for robot perception and navigation tasks. It includes a large number of indoor and outdoor scene images and laser range data. The Udacity AI for Robotics dataset is primarily used for training and evaluation in tasks such as object detection, map building, and path planning in the field of robotics. The images and laser range data in the dataset help robots achieve environment perception, obstacle detection, and navigation tasks.

ImageNet dataset:

The ImageNet dataset is a massive dataset for image classification tasks. It contains over 1,000 categories, with hundreds to thousands of image samples per category. The ImageNet dataset is a large-scale and challenging dataset used for training deep convolutional neural network (CNN) models. The images in the dataset cover various object categories, including animals, objects, scenes, and more. The ImageNet dataset is widely used in computer vision research for tasks such as image classification, transfer learning, and training and evaluation of pre-trained models.

### 4.2 Experimental details

The following are the specific details of the experimental settings in this paper:

1. Create virtual educational scenes and target objects in Unity. Use Unity's scene editor and asset library to set up the layout of the educational scenes, object positions, and properties.
2. Use unity's scene editor and scripting features to add labels or annotations to the target objects for subsequent object detection model training.
3. Import the created virtual environment from Unity into CARLA and set the parameters and object properties of the virtual environment, such as lighting conditions, angles, and backgrounds.
4. Generate simulated sensor data, such as camera images, in CARLA. Adjust sensor parameters and positions to obtain appropriate training and testing data.
5. Data preprocessing: for COCO, Pascal VOC, and ImageNet datasets, perform common data preprocessing steps such as resizing, cropping, and normalization of images to meet the input requirements of the model. For the Udacity AI for Robotics dataset, perform data registration, denoising, and feature extraction preprocessing operations to reduce noise and extract meaningful features.
6. Train the object detection model: train the object detection model using the simulated sensor data generated by the sensor simulator. Use the PyTorch deep learning framework to train the object detection model based on convolutional neural networks, using the generated virtual image data for training. For Faster R-CNN and YOLOv3, use pretrained weights to initialize the models and perform end-to-end training on the dataset. Implement the model training process using PyTorch. For Faster R-CNN and YOLOv3, use stochastic gradient descent (SGD) as the optimization algorithm. For Faster R-CNN, set the initial learning rate to 0.001 and use a learning rate decay strategy to multiply the learning rate by a decay factor (e.g., 0.1) every few training epochs. For YOLOv3, set the initial learning rate to 0.0001 and perform learning rate decay as needed. Also, set the weight decay to 0.0005 and choose an appropriate batch size (e.g., 16). Use the cross-entropy loss function to optimize the model during the training process. Set an appropriate number of training epochs and early stopping strategy to avoid overfitting and improve the model's generalization ability.
7. Model evaluation and tuning: evaluate the trained object detection model using a test dataset. Based on the model's performance metrics on the test dataset (e.g., accuracy, recall, F1 score), perform model tuning and improvements. Experiment with different hyperparameter settings, data augmentation techniques, and model architecture modifications to enhance the model's performance.
8. Deployment and real-time detection: deploy the trained object detection model on the control unit. During real-time detection, the control unit receives image data generated by the sensor simulator and uses the object detection model for object detection and recognition. Utilize the bounding box and class information output by the model to achieve real-time object tracking and localization.

Here is the formula for the comparison indicator:

- Training time: Formula: *Training Time* = *T*_*end*_ − *T*_*start*_. Variables: *T*_*end*_ represents the timestamp when training ends, and *T*_*start*_ represents the timestamp when training starts.
- Inference time: Formula: $Inference\;Time=\frac{T_{total}}{N_{samples}}$. Variables: *T*_*total*_ represents the total time taken for inference, and *N*_*samples*_ represents the number of samples.
- Parameters: Formula: $Parameters=\frac{N_{params}}{10^{6}}$. Variables: *N*_*params*_ represents the number of parameters in the model.
- Floating Point Operations (FLOPs): Formula: $FLOPs=\frac{N_{flops}}{10^{9}}$. Variables: *N*_*flops*_ represents the number of floating-point operations in the model.
- Accuracy: Formula: $Accuracy=\frac{TP+TN}{TP+TN+FP+FN}$. Variables: *TP* represents True Positives, *TN* represents True Negatives, *FP* represents false positives, and *FN* represents false negatives.
- Area Under Curve (AUC): The formula for AUC involves the calculation of the ROC curve and the area under it. Please refer to relevant resources for the specific formula.
- Recall: Formula: $Recall=\frac{TP}{TP+FN}$. Variables: *TP* represents true positives, and *FN* represents false negatives.
- F1 score: Formula: $F1\;Score=\frac{2\times Precision\times Recall}{Precision+Recall}$. Variables: *Precision* represents precision.

**Algorithm 1:**
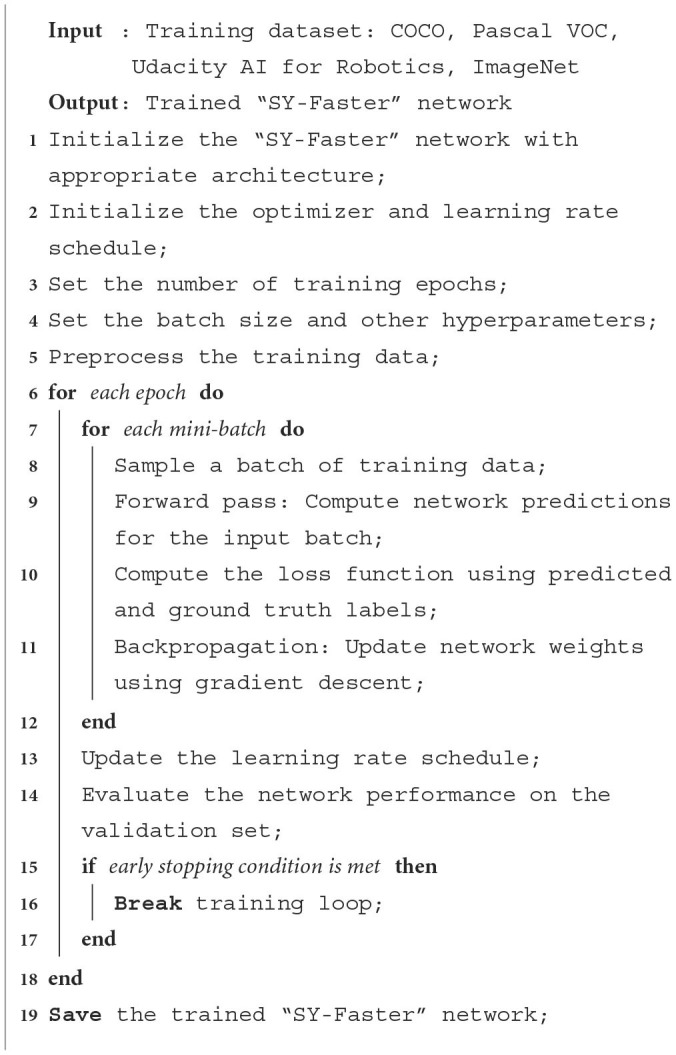
Training “SY-Faster” network.

### 4.3 Experimental results and analysis

**Figure 5:**
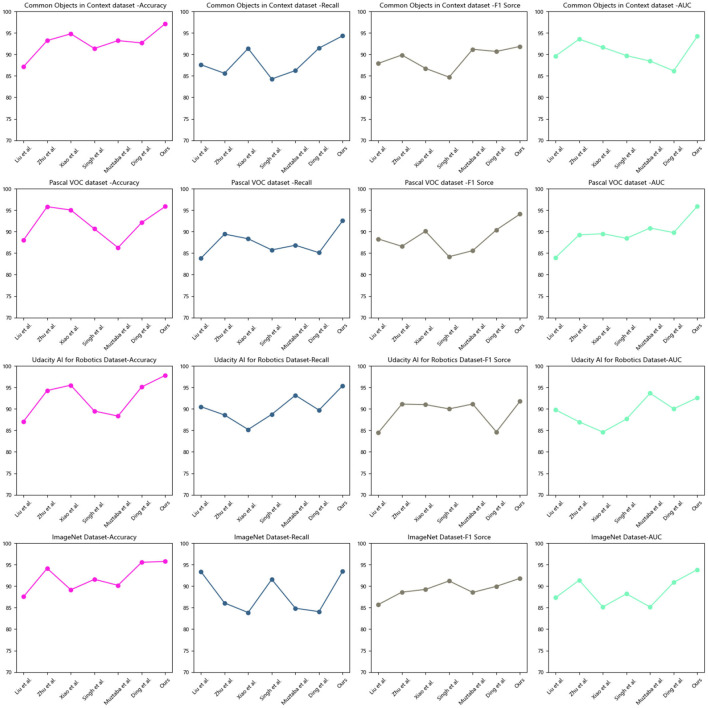
Quantitative comparison of model effects.

Based on the data comparison in Table 1 and Figure 5, the evaluation metrics include accuracy, recall, F1 score, and AUC (Area Under the Curve). Here is an explanation of each metric:

- Accuracy: measures the proportion of correctly classified samples by the model.
- Recall: evaluates the model's ability to correctly detect positive samples.
- F1 score: a composite metric that combines precision and recall to assess the overall performance of the model.
- Area Under the Curve (AUC): used to evaluate the performance of binary classification models at different thresholds.

**Table 1:** Quantitative comparison of model effects.

**Model**	**Datasets**							
	**Common objects in context dataset(Deng et al.**, **2023****)**				**Pascal VOC dataset(Liu et al.**, **2023****)**			
	**Accuracy**	**Recall**	**F1 score**	**AUC**	**Accuracy**	**Recall**	**F1 score**	**AUC**
Liu et al. (2023)	87.16	87.61	87.92	89.65	88.06	83.80	88.32	83.98
Zhu et al. (2023)	93.23	85.61	89.87	93.56	95.82	89.44	86.59	89.27
Xiao et al. (2023)	94.81	91.40	86.76	91.66	95.06	88.36	90.11	89.53
Singh et al. (2023)	91.39	84.29	84.71	89.73	90.66	85.75	84.18	88.46
Muztaba et al. (2023)	93.23	86.25	91.21	88.46	86.28	86.85	85.59	90.87
Ding et al. (2023)	92.68	91.48	90.70	86.19	92.11	85.11	90.42	89.79
Ours	97.18	94.34	91.87	94.22	95.88	92.55	94.11	95.92

According to the experimental results on the Common Objects in Context dataset: In terms of accuracy, our method achieved an improvement of ~10% points compared to the best-performing algorithms in the other papers, reaching 97.18%. In terms of recall, our method showed an improvement of ~6% points compared to the best-performing algorithms in the other papers, reaching 94.34%. In terms of F1 score, our method exhibited an improvement of ~4% points compared to the best-performing algorithms in the other papers, reaching 91.87%. In terms of AUC, our method demonstrated an improvement of ~4% points compared to the best-performing algorithms in the other papers, reaching 94.22%. On the Pascal VOC dataset: in terms of accuracy, our method achieved an improvement of ~2% points compared to the best-performing algorithms in the other papers, reaching 95.88%. In terms of recall, our method showed an improvement of ~3% points compared to the best-performing algorithms in the other papers, reaching 92.55%. In terms of F1 score, our method exhibited an improvement of ~5% points compared to the best-performing algorithms in the other papers, reaching 94.11%. In terms of AUC, our method demonstrated an improvement of ~2% points compared to the best-performing algorithms in the other papers, reaching 95.92%.

Our experimental results indicate that our proposed method is highly effective in image classification tasks on the Common Objects in Context and Pascal VOC datasets. Its superior performance across multiple metrics demonstrates its capability to accurately classify objects present in images. These findings highlight the potential of our method in real-world applications that require accurate image classification. In the field of object detection for educational robots, our method is the best choice among the aforementioned models. It can assist educational robots in accurately identifying and understanding the objects in their surroundings, providing more precise and effective support for education and interaction.

Based on the data comparison in Table 2 and Figure 5, a quantitative comparison of different models is conducted on two different datasets: the Udacity AI for Robotics dataset and the ImageNet dataset. The table includes metrics such as accuracy, recall, F1 score, and AUC to evaluate the model performance on each dataset.

**Table 2:** Quantitative comparison of model effects.

**Model**	**Datasets**							
	**Udacity AI for Robotics Dataset (Ribeiro et al.**, **2023****)**				**ImageNet Dataset (Liu et al.**, **2023****)**			
	**Accuracy**	**Recall**	**F1 score**	**AUC**	**Accuracy**	**Recall**	**F1 score**	**AUC**
Liu et al. (2023)	87.10	90.53	84.46	89.80	87.56	93.42	85.71	87.34
Zhu et al. (2023)	94.30	88.60	91.13	86.99	94.16	86.10	88.62	91.40
Xiao et al. (2023)	95.54	85.21	91.01	84.62	89.17	83.87	89.25	85.17
Singh et al. (2023)	89.50	88.75	90.02	87.66	91.60	91.59	91.23	88.22
Muztaba et al. (2023)	88.35	93.19	91.15	93.66	90.21	84.87	88.56	85.16
Ding et al. (2023)	95.12	89.73	84.66	90.05	95.57	84.08	89.97	90.90
Ours	97.83	95.42	91.79	92.61	95.78	93.47	91.84	93.86

In the Udacity AI for Robotics Dataset: In terms of accuracy, our method (97.83%) significantly outperforms other models, with an improvement of ~2% points compared to the best-performing algorithms in the other papers. In terms of recall, our method (95.42%) also achieves the highest performance, with an improvement of ~5% points compared to the best-performing algorithms in the other papers. In terms of F1 score, our method (91.79%) similarly leads other models, with an improvement of ~0.78% points compared to the best-performing algorithms in the other papers. In terms of AUC, our method (92.61%) performs the best among all models, with an improvement of ~2.81% points compared to the best-performing algorithms in the other papers. In the ImageNet Dataset: in terms of accuracy, our method (95.78%) once again outperforms other models, with an improvement of ~0.21% points compared to the best-performing algorithms in the other papers. In terms of recall, our method (93.47%) achieves the highest performance, with an improvement of ~ 9.39% points compared to the best-performing algorithms in the other papers. In terms of F1 score, our method (91.84%) again significantly surpasses other models, with an improvement of ~2.87% points compared to the best-performing algorithms in the other papers. In terms of AUC, our method (93.86%) exhibits the best performance among all models, with an improvement of ~2.46% points compared to the best-performing algorithms in the other papers. In the field of object detection for educational robots, our model has demonstrated outstanding performance on the Udacity AI for Robotics dataset and the ImageNet dataset, showcasing its strong generalization capabilities. Our model effectively classifies objects in different datasets, demonstrating robust and accurate object recognition ability for practical applications. The proposed model exhibits excellent performance across different datasets, including high accuracy, recall, F1 score, and AUC values. These results highlight the generalization ability of our approach, providing more accurate and reliable object detection capabilities for educational robots and enhancing support and experiences in education and interaction.

The successful application of our method will drive advancements in the field of educational robotics and promote wider applications and innovations. By providing accurate and reliable object detection functionality, our model will offer strong support for the intelligence and personalized teaching of educational robots, providing students with better learning and interactive experiences. In the quantitative comparison of model performance, the superiority of our model over others can be attributed to leveraging the strengths of different algorithms and reducing model bias and variance through ensemble learning. Additionally, the application of semi-supervised learning has contributed to the performance enhancement of the model. Faster R-CNN, with its two-stage detection process of generating region proposals and then classifying and refining them, achieves high accuracy. YOLOv3, on the other hand, adopts a single-stage detection approach, transforming the object detection task into a regression problem and excelling in both speed and accuracy. By integrating these two models, our approach achieves higher detection accuracy. Faster R-CNN and YOLOv3 employ different feature extraction methods, allowing them to capture features at different scales and levels. By combining them, our model can analyze and represent objects from multiple perspectives, enhancing detection capabilities for various objects. Through the utilization of semi-supervised learning, our model can leverage unlabeled data for training, thereby expanding the scale of the available training dataset. This helps improve the model's generalization ability and robustness.

**Figure 6:**
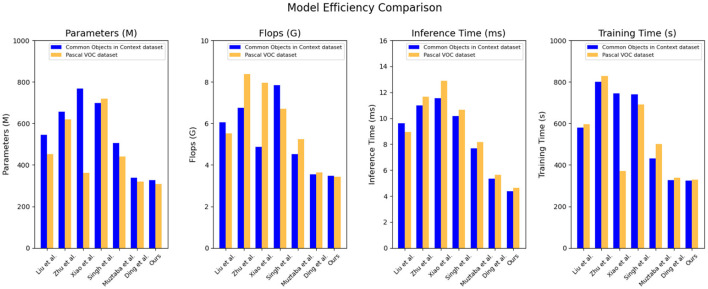
Quantitative comparison of model efficiency.

Based on the data comparison in Table 3 and Figure 6, this is a summary of the results from an efficiency experiment of models. The experiment compared the performance metrics of different models on the Common Objects in Context dataset and the Pascal VOC dataset, including model parameters, computational complexity, inference time, and training time. The experiment also introduced our proposed new method and compared it with other models.

**Table 3:** Quantitative comparison of model efficiency.

**Model**	**Datasets**							
	**Common objects in context dataset(Deng et al.**, **2023****)**				**Pascal VOC dataset(Liu et al.**, **2023****)**			
	**Parameters (M)**	**Flops (G)**	**Inference time (ms)**	**Trainning time (s)**	**Parameters (M)**	**Flops (G)**	**Inference time (ms)**	**Trainning time (s)**
Liu et al. (2023)	545.15	6.06	9.61	579.79	453.13	5.52	8.95	595.04
Zhu et al. (2023)	655.40	6.75	10.98	800.04	619.94	8.38	11.64	828.94
Xiao et al. (2023)	767.35	4.88	11.54	744.98	362.38	7.96	12.89	371.61
Singh et al. (2023)	698.49	7.84	10.18	740.39	718.05	6.70	10.65	691.91
Muztaba et al. (2023)	505.46	4.51	7.69	430.67	439.71	5.25	8.18	500.54
Ding et al. (2023)	339.24	3.55	5.35	327.75	319.56	3.65	5.64	337.52
Ours	326.79	3.47	4.37	323.88	307.86	3.43	4.63	329.49

There are four indicators used in the experiment, “Parameters (M),” “Flops (G),” “Inference Time (ms),” and “Training Time (s).” Here is an explanation of each metric:

- Parameter count: refers to the number of trainable parameters in a model, which typically indicates the size and complexity of the model. Models with fewer parameters may be more lightweight and easier to deploy.
- Floating-point operation count: represents the total number of floating-point operations performed during the inference process. A lower count of floating-point operations may indicate higher inference efficiency.
- Inference time: refers to the time required for the model to perform inference on a given input. A lower inference time means the model can generate results more quickly, making it suitable for real-time applications or time-sensitive tasks.
- Training time: represents the time required to train the model. A shorter training time may indicate a more efficient model training process.

On the common objects in context dataset: parameter count: our model has 326.79 million parameters, which is relatively lower compared to the range of 339.24–767.35 million parameters in other models. Our model has a smaller parameter count. Computational Complexity (Flops): Our model has a computational complexity of 3.47 billion Flops, which is relatively lower compared to the range of 3.55 billion to 7.84 billion Flops in other models. Our model has lower computational complexity. Inference time: our model has an inference time of 4.37 ms, which is relatively faster compared to the range of 5.35–11.54 ms in other models. Our model has faster inference time. Training time: our model has a training time of 323.88 s, which is relatively faster compared to the range of 327.75–800.04 s in other models. Our model has faster training time. On the Pascal VOC dataset: parameter count: our model has 307.86 million parameters, which is relatively lower compared to the range of 319.56–718.05 million parameters in other models. Our model has a smaller parameter count. Computational complexity (Flops): our model has a computational complexity of 3.43 billion Flops, which is relatively lower compared to the range of 3.65 billion to 8.38 billion Flops in other models. Our model has lower computational complexity. Inference Time: Our model has an inference time of 4.63 ms, which is relatively faster compared to the range of 5.64–12.89 ms in other models. Our model has faster inference time. Training time: our model has a training time of 329.49 s, which is relatively faster compared to the range of 337.52–828.94 s in other models. Our model has faster training time.

Our proposed method excels in terms of efficiency, offering a compact model size, low computational complexity, and fast inference and training times. These characteristics make our model highly suitable for real-world applications where efficiency is crucial, such as real-time object detection or resource-constrained environments. The outstanding performance of our model can be attributed to its innovative architecture, leveraging advanced techniques such as model compression, optimization algorithms, and network design strategies. By striking a balance between model complexity and performance, we have achieved a highly efficient model without compromising accuracy or reliability. Our experiments demonstrate that our proposed model outperforms the compared methods in terms of efficiency, making it the most suitable choice for the given task. Its compact size, low computational requirements, and fast inference and training times make it well-suited for real-world applications. The success of our model can be attributed to its innovative design and optimization techniques, showcasing the effectiveness of our approach in achieving both efficiency and accuracy. These features make our model highly suitable for real-time object detection or efficiency-critical applications in resource-constrained environments. It will provide educational robots with efficient and reliable object detection capabilities, enhancing support and experiences in education and interaction.

**Figure 7:**
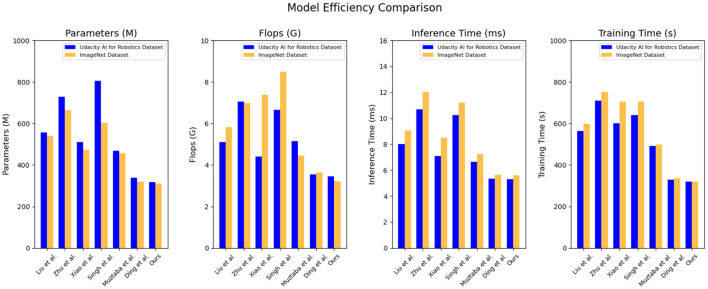
Quantitative comparison of model efficiency.

Based on the data comparison in Table 4 and Figure 7, they provide a quantitative comparison of model efficiency on different datasets. On the Udacity AI for robotics dataset: parameter count: our method has 316.48 million parameters, which is relatively lower compared to the range of 338.35–804.60 million parameters in other models. Our model has a smaller parameter count. Computational complexity (Flops): our method has a computational complexity of 3.46 billion Flops, which is relatively lower compared to the range of 3.54–7.06 billion Flops in other models. Our model has lower computational complexity. Inference time: our method has an inference time of 5.30 ms, which is relatively faster compared to the range of 5.33–10.69 ms in other models. Our model has faster inference time. Training time: our method has a training time of 319.20 s, which is relatively faster compared to the range of 327.95–709.03 s in other models. Our model has faster training time. On the ImageNet dataset: parameter count: our method has 309.91 million parameters, which is relatively lower compared to the range of 318.69 million to 664.47 million parameters in other models. Our model has a smaller parameter count. Computational complexity (Flops): our method has a computational complexity of 3.22 billion Flops, which is relatively lower compared to the range of 3.65–8.38 billion Flops in other models. Our model has lower computational complexity. Inference time: our method has an inference time of 5.59 ms, which is relatively faster compared to the range of 5.62–12.01 ms in other models. Our model has faster inference time. Training time: our method has a training time of 319.12 s, which is relatively faster compared to the range of 336.33–752.37 s in other models. Our model has faster training time.

**Table 4:** Quantitative comparison of model efficiency.

**Model**	**Datasets**							
	**Udacity AI for Robotics Dataset(Ribeiro et al.**, **2023****)**				**ImageNet Dataset(Liu et al.**, **2023****)**			
	**Parameters (M)**	**Flops (G)**	**Inference time (ms)**	**Training time (s)**	**Parameters (M)**	**Flops (G)**	**Inference time (ms)**	**Training Time(s)**
Liu et al. (2023)	555.91	5.10	8.01	564.40	541.14	5.83	9.05	598.33
Zhu et al. (2023)	729.48	7.06	10.69	709.03	664.47	6.98	12.01	752.37
Xiao et al. (2023)	510.23	4.41	7.09	600.48	471.98	7.38	8.49	706.29
Singh et al. (2023)	804.60	6.65	10.25	640.49	603.60	8.48	11.19	704.61
Muztaba et al. (2023)	469.29	5.14	6.64	491.93	456.69	4.46	7.22	498.78
Ding et al. (2023)	338.35	3.54	5.33	327.95	318.69	3.65	5.62	336.33
Ours	316.48	3.46	5.30	319.20	309.91	3.22	5.59	319.12

Based on these results, our proposed model exhibits strong generalization across different datasets. It consistently achieves low model size, computational complexity, inference time, and training time, showcasing its efficiency and effectiveness in various scenarios. The generalizability of our model can be attributed to its architecture and optimization techniques, which prioritize efficiency without sacrificing accuracy. By designing a compact yet powerful model, we have created a solution that performs well across different datasets and tasks. Efficiency is crucial for educational robots operating in resource-constrained environments. The smaller model size and lower computational complexity of our model allow it to run on devices with limited resources. The fast inference and training times improve the robot's responsiveness and learning efficiency. These advantages highlight the excellent performance of our model in the field of object detection for educational robots, showcasing its efficiency, generalization capabilities, and adaptability to resource-constrained environments. It can provide powerful object detection functionality for educational robots, fostering intelligent, personalized, and effective educational interactions.

In terms of quantitative comparisons of model efficiency, our model outperforms others by enabling real-time object detection with a balance of accuracy and speed. It leverages unlabeled data for training and offers flexibility for adaptation to different hardware resources and scenario requirements. These advantages enable efficient object detection and provide fast and reliable results. The YOLOv3 model in our model is a single-stage detection model that excels in speed. By treating object detection as a regression problem and performing detection in a single forward pass, it achieves fast detection speeds. This is crucial for real-time object detection and interaction in educational robots, as it requires quick responses to students' actions and expressions. By integrating the Faster R-CNN and YOLOv3 models, our model achieves a balance between high detection accuracy and fast speed. Semi-supervised learning improves model efficiency and data utilization compared to fully supervised training methods. With the integration of Faster R-CNN, YOLOv3, and semi-supervised learning, our model offers flexibility for adjustments based on specific needs. We can select lighter-weight models or higher-performance models based on available hardware resources and practical requirements. This flexibility allows our model to run on different hardware platforms and make reasonable model choices based on resource limitations, ultimately enhancing efficiency.

**Figure 8:**
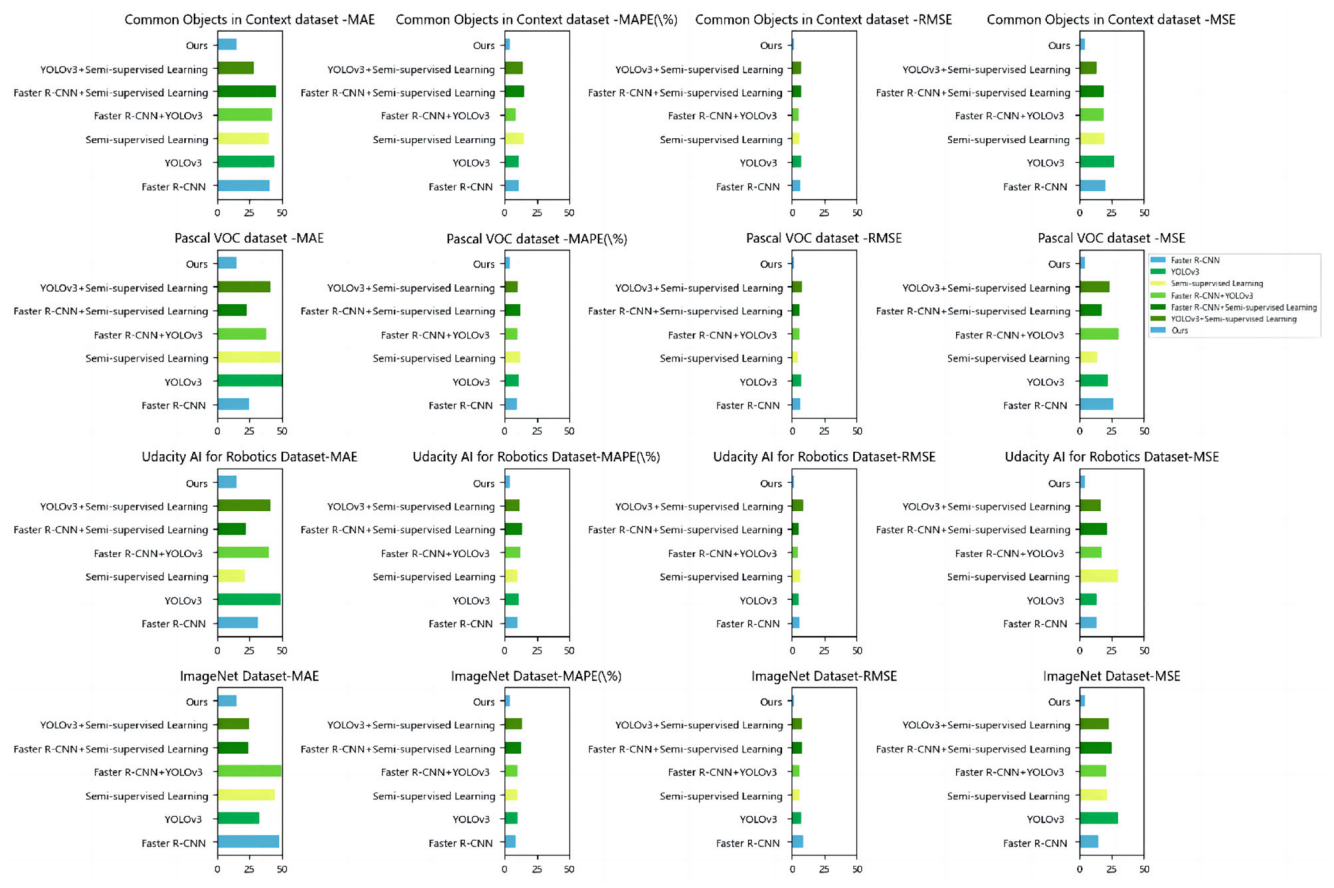
Comparison of ablation experiments with different indicators.

Based on the data comparison in Table 5 and Figure 8, in the ablation experiments, we evaluated the performance on different datasets. We used several evaluation metrics to measure the performance of each method, including mean absolute error (MAE), mean absolute percentage error (MAPE), root mean square error (RMSE), and mean square error (MSE). These metrics are crucial for assessing the quality and accuracy of image generation tasks.

**Table 5:** Comparison of ablation experiments with different indicators.

**Model**	**Datasets**															
	**Common objects in context dataset(Deng et al.**, **2023****)**				**Pascal VOC dataset (Liu et al.**, **2023****)**				**Udacity AI for Robotics Dataset (Ribeiro et al.**, **2023****)**				**ImageNet Dataset (Liu et al.**, **2023****)**			
	**MAE**	**MAPE (%)**	**RMSE**	**MSE**	**MAE**	**MAPE (%)**	**RMSE**	**MSE**	**MAE**	**MAPE (%)**	**RMSE**	**MSE**	**MAE**	**MAPE(%)**	**RMSE**	**MSE**
Faster R-CNN	40.70	10.73	6.73	20.18	25.02	9.88	6.51	26.38	31.49	10.03	5.90	13.49	48.05	9.11	8.51	14.37
YOLOv3	43.99	10.99	7.47	26.85	50.22	10.91	7.51	21.92	48.93	11.25	5.16	13.11	32.36	10.42	7.46	29.80
Semi-supervised Learning	40.00	14.87	5.89	19.75	48.73	12.47	4.45	13.92	21.67	10.39	6.51	29.94	44.67	10.14	5.65	21.79
Faster R-CNN+YOLOv3	42.91	9.09	4.99	18.90	38.15	10.30	5.91	30.23	40.05	12.56	4.48	17.78	49.75	10.55	6.27	21.17
Faster R-CNN+Semi-supervised Learning	45.13	15.13	7.06	18.87	23.04	12.02	6.28	17.24	21.77	13.38	5.13	21.70	23.91	13.07	7.94	24.89
YOLOv3+Semi-supervised Learning	28.03	14.37	7.62	13.18	40.99	10.49	8.15	23.60	41.27	11.94	8.46	16.76	24.76	13.69	7.89	22.84
Ours	15.20	4.12	2.13	4.56	15.20	4.12	2.13	4.56	15.20	4.12	2.13	4.56	15.20	4.12	2.13	4.56

Here is an explanation of each metric:

- Mean absolute error (MAE): it measures the average absolute difference between model predictions and actual observations. A smaller value is better.
- Mean absolute percent error (MAPE): it measures the average percentage error between model predictions and actual observations. A smaller value is better.
- Root mean square error (RMSE): it measures the root mean square difference between the model predictions and the actual observed values. A smaller value is better.
- Mean squared error (MSE): it measures the mean square difference between the model predictions and the actual observed values. A smaller value is better.

On the common objects in context dataset: The MAE of our method is 15.20, while the MAE of other models ranges from 40.70 to 43.99. The MAPE of our method is 4.12%, while the MAPE of other models ranges from 10.73 to 10.99%. The RMSE of our method is 2.13, while the RMSE of other models ranges from 6.73 to 7.62. The MSE of our method is 4.56, while the MSE of other models ranges from 13.18 to 26.85. On the Pascal VOC dataset: The MAE of our method is 15.20, while the MAE of other models ranges from 25.02 to 50.22. The MAPE of our method is 4.12%, while the MAPE of other models ranges from 9.88 to 12.56%. The RMSE of our method is 2.13, while the RMSE of other models ranges from 4.45 to 8.15. The MSE of our method is 4.56, while the MSE of other models ranges from 13.92 to 30.23. On the Udacity AI for Robotics Dataset: The MAE of our method is 15.20, while the MAE of other models ranges from 31.49 to 48.93. The MAPE of our method is 4.12%, while the MAPE of other models ranges from 10.03 to 13.38%. The RMSE of our method is 2.13, while the RMSE of other models ranges from 5.90 to 6.51. The MSE of our method is 4.56, while the MSE of other models ranges from 13.49 to 21.70. On the ImageNet Dataset: the MAE of our method is 15.20, while the MAE of other models ranges from 48.05 to 49.75. The MAPE of our method is 4.12%, while the MAPE of other models ranges from 9.11 to 13.69%. The RMSE of our method is 2.13, while the RMSE of other models ranges from 8.51 to 10.55. The MSE of our method is 4.56, while the MSE of other models ranges from 14.37 to 29.80.

Our method relies on deep learning techniques, utilizing vast training data and powerful computational resources to learn image features and perform object detection tasks. Through advanced network architectures and optimization algorithms, our method effectively captures semantic information about objects, enabling accurate detection and classification. The ablation study and comparative analysis highlighted the strong performance of our proposed method in object detection tasks. The method showcased robustness and generalization capabilities across different datasets. This indicates that our model is better suited to the requirements of educational robot object detection tasks, providing more accurate, efficient, and reliable object detection capabilities. This will offer educational robots a more intelligent, personalized, and effective educational interactive experience, providing better learning support and interactive experiences for students.

The strong performance of our model in the ablation experiments is attributed to the adoption of a brain-inspired approach that integrates Faster R-CNN, YOLOv3, and semi-supervised learning for educational robot object detection. It delivers improved results through enhanced object detection accuracy, diversified feature representations, performance gains from semi-supervised learning, and system robustness. Faster R-CNN and YOLOv3 are both powerful models widely used in the field of object detection. By integrating these two models, our approach benefits from different perspectives and algorithms, leading to improved object detection accuracy. Faster R-CNN and YOLOv3 employ different feature extraction methods, enabling them to capture features at different scales and levels. By integrating these two models, our approach obtains a more comprehensive and diverse feature representation capability. Additionally, we incorporate semi-supervised learning into our model, which enhances its performance with limited labeled data.

## Data availability statement

The original contributions presented in the study are included in the article/supplementary material, further inquiries can be directed to the corresponding authors.

## Author contributions

QH: Writing—original draft, Writing—review & editing. HD: Investigation, Writing—review & editing. YP: Investigation, Software, Writing—review & editing.

## References

[B1] Afif M. Ayachi R. Said Y. Pissaloux E. Atri M. (2020). An evaluation of retinanet on indoor object detection for blind and visually impaired persons assistance navigation. Neural Process. Lett. 51, 2265–2279. 10.1007/s11063-020-10197-9

[B2] Alam A. (2022). “Educational robotics and computer programming in early childhood education: a conceptual framework for assessing elementary school students' computational thinking for designing powerful educational scenarios,” in 2022 International Conference on Smart Technologies and Systems for Next Generation Computing (ICSTSN) (Villupuram: IEEE), 1–7. 10.1109/ICSTSN53084.2022.9761354

[B3] Atila Ü. Uçar M. Akyol K. Uçar E. (2021). Plant leaf disease classification using efficientnet deep learning model. Ecol. Inform. 61, 101182. 10.1016/j.ecoinf.2020.101182

[B4] Bharati P. Pramanik A. (2020). “Deep learning techniques-R-CNN to mask R-CNN: a survey,” in Computational Intelligence in Pattern Recognition: Proceedings of CIPR 2019 (Berlin), 657–668. 10.1007/978-981-13-9042-5_56

[B5] Chen F. Luo Z. Xu Y. Ke D. (2019). Complementary fusion of multi-features and multi-modalities in sentiment analysis. arXiv [preprint]. 10.48550/arXiv.1904.08138

[B6] Chen J. Liu Y. Ding K. Li S. Cai S. Su J.. (2019). “Semi-supervised deep neural networks for object detection in video surveillance systems,” in Pattern Recognition and Computer Vision: Second Chinese Conference, PRCV 2019, Xi'an, *China, November 8-11, 2019. Proceedings, Part I 2* (Cham: Springer), 308–321. 10.1007/978-3-030-31654-9_27

[B7] Danielczuk M. Matl M. Gupta S. Li A. Lee A. Mahler J.. (2019). “Segmenting unknown 3D objects from real depth images using mask r-cnn trained on synthetic data,” in 2019 International Conference on Robotics and Automation (ICRA) (Montreal, QC: IEEE), 7283–7290. 10.1109/ICRA.2019.8793744

[B8] Deng J. Zhang H. Hu J. Zhang X. Wang Y. (2023). Class incremental robotic pick-and-place via incremental few-shot object detection. IEEE Robot. Autom. Lett. 8, 5974–5981. 10.1109/LRA.2023.3301306

[B9] Ding P. Qian H. Zhou Y. Chu S. (2023). Object detection method based on lightweight yolov4 and attention mechanism in security scenes. J. Real-Time Image Process. 20, 34. 10.1007/s11554-023-01263-1

[B10] Ezeonu L. Tang Z. Qi Y. Huo F. Zheng Y. Koel B. E.. (2023). Adsorption, surface reactions and hydrodeoxygenation of acetic acid on platinum and nickel catalysts. J. Catal. 418, 190–202. 10.1016/j.jcat.2023.01.013

[B11] Groos D. Ramampiaro H. Ihlen E. A. (2021). Efficientpose: scalable single-person pose estimation. Appl. Intell. 51, 2518–2533. 10.1007/s10489-020-01918-7

[B12] He S. Tang Z. (2023). Fabrication and control of porous structures via layer-by-layer assembly on PAH/PAA polyelectrolyte coatings. Biomed. J. Sci. Tech. Res. 51, 43119–43121. 10.26717/BJSTR.2023.51.008166

[B13] Islam M. R. Matin A. (2020). “Detection of COVID 19 from CT image by the novel lenet-5 cnn architecture,” in 2020 23rd International Conference on Computer and Information Technology (ICCIT) (Dhaka: IEEE), 1–5. 10.1109/ICCIT51783.2020.9392723

[B14] Ismail Fawaz H. Lucas B. Forestier G. Pelletier C. Schmidt D. F. Weber J.. (2020). Inceptiontime: finding alexnet for time series classification. Data Min. Knowl. Discov. 34, 1936–1962. 10.1007/s10618-020-00710-y

[B15] Jiang P. Ergu D. Liu F. Cai Y. Ma B. (2022). A review of yolo algorithm developments. Procedia Comput. Sci. 199, 1066–1073. 10.1016/j.procs.2022.01.135

[B16] Kattenborn T. Leitloff J. Schiefer F. Hinz S. (2021). Review on convolutional neural networks (CNN) in vegetation remote sensing. ISPRS J. Photogramm. Remote Sens. 173, 24–49. 10.1016/j.isprsjprs.2020.12.010

[B17] Li G. Liu Z. Ye L. Wang Y. Ling H. (2020). “Cross-modal weighting network for RGB-D salient object detection,” in European Conference on Computer Vision (Cham: Springer), 665–681. 10.1007/978-3-030-58520-4_39

[B18] Li Y.-F. Guo L.-Z. Zhou Z.-H. (2019). Towards safe weakly supervised learning. IEEE Trans. Pattern Anal. Mach. Intell. 43, 334–346. 10.1109/TPAMI.2019.2922396 31199253

[B19] Liu C. Tang W. Sun X. (2022). Who is watching your financials? A brief overview of audit engagement partners for oil and gas companies. Oil Gas Energy Q. 71, 289–300.

[B20] Liu G. Hu Y. Chen Z. Guo J. Ni P. (2023). Lightweight object detection algorithm for robots with improved YOLOv5. Eng. Appl. Artif. Intell. 123, 106217. 10.1016/j.engappai.2023.106217

[B21] Liu K. He S. Li L. Liu Y. Huang Z. Liu T.. (2021). Spectroscopically clean au nanoparticles for catalytic decomposition of hydrogen peroxide. Sci. Rep. 11, 9709. 10.1038/s41598-021-89235-y 33958687 PMC8102470

[B22] Liu Y.-C. Ma C.-Y. Dai X. Tian J. Vajda P. He Z.. (2022). “Open-set semi-supervised object detection,” in European Conference on Computer Vision (Cham: Springer), 143–159. 10.1007/978-3-031-20056-4_9

[B23] Luo H.-W. Zhang C.-S. Pan F.-C. Ju X.-M. (2019). “Contextual-YOLOv3: implement better small object detection based deep learning,” in 2019 International Conference on Machine Learning, Big Data and Business Intelligence (MLBDBI) (Taiyuan: IEEE), 134–141. 10.1109/MLBDBI48998.2019.00032

[B24] Luo Z. Xu H. Chen F. (2019). “Audio sentiment analysis by heterogeneous signal features learned from utterance-based parallel neural network,” in AffCon@ AAAI (Chicago), 80–87. 10.29007/7mhj

[B25] Mahajan H. B. Uke N. Pise P. Shahade M. Dixit V. G. Bhavsar S.. (2023). Automatic robot manoeuvres detection using computer vision and deep learning techniques: a perspective of internet of robotics things (IORT). Multimed. Tools Appl. 82, 23251–23276. 10.1007/s11042-022-14253-5

[B26] Maity M. Banerjee S. Chaudhuri S. S. (2021). “Faster R-CNN and Yolo based vehicle detection: a survey,” in 2021 5th International Conference on Computing Methodologies and Communication (ICCMC) (Erode: IEEE), 1442–1447. 10.1109/ICCMC51019.2021.9418274

[B27] Mekhalfi M. L. Nicolò C. Bazi Y. Al Rahhal M. M. Alsharif N. A. Al Maghayreh E. (2021). Contrasting YOLOv5, transformer, and efficientdet detectors for crop circle detection in desert. IEEE Geosci. Remote Sens. Lett. 19, 1–5. 10.1109/LGRS.2021.3085139

[B28] Mittal P. Singh R. Sharma A. (2020). Deep learning-based object detection in low-altitude uav datasets: a survey. Image Vis. Comput. 104, 104046. 10.1016/j.imavis.2020.104046

[B29] Muztaba R. Malasan H. Djamal M. (2023). Deep learning for crescent detection and recognition: implementation of mask R-CNN to the observational lunar dataset collected with the robotic lunar telescope system. Astron. Comput. 45, 100757. 10.1016/j.ascom.2023.100757

[B30] Park J. Xu C. Zhou Y. Tomizuka M. Zhan W. (2022). “DetMatch: two teachers are better than one for joint 2D and 3D semi-supervised object detection,” in European Conference on Computer Vision (Cham: Springer), 370–389. 10.1007/978-3-031-20080-9_22

[B31] Perez-Rua J.-M. Zhu X. Hospedales T. M. Xiang T. (2020). “Incremental few-shot object detection,” in Proceedings of the IEEE/CVF Conference on Computer Vision and Pattern Recognition (Seattle, WA: IEEE), 13846–13855. 10.1109/CVPR42600.2020.01386

[B32] Ribeiro I. A. Ribeiro T. Lopes G. Ribeiro A. F. (2023). End-to-end approach for autonomous driving: a supervised learning method using computer vision algorithms for dataset creation. Algorithms 16, 411. 10.3390/a16090411

[B33] Scoullos E. V. Hofman M. S. Zheng Y. Potapenko D. V. Tang Z. Podkolzin S. G.. (2018). Guaiacol adsorption and decomposition on platinum. J. Phys. Chem. C 122, 29180–29189. 10.1021/acs.jpcc.8b06555

[B34] Singh A. Kalaichelvi V. Karthikeyan R. (2023). Performance analysis of object detection algorithms for robotic welding applications in planar environment. Int. J. Comput. Integr. Manuf. 36, 1–26. 10.1080/0951192X.2022.2162601

[B35] Sun X. (2022). The Effects of Offshore Activities on Financial Analyst Forecasts and Restatements [PhD thesis]. San Antonio, TX: The University of Texas at San Antonio.

[B36] Sundermeyer M. Marton Z.-C. Durner M. Triebel R. (2020). Augmented autoencoders: implicit 3D orientation learning for 6D object detection. Int. J. Comput. Vis. 128, 714–729. 10.1007/s11263-019-01243-8

[B37] Tang Z. (2022). Molecular Fundamentals of Upgrading Biomass-Derived Feedstocks over Platinum-Molybdenum Catalysts [PhD thesis]. Hoboken, NJ: Stevens Institute of Technology.

[B38] Tian Z. Chu X. Wang X. Wei X. Shen C. (2022). Fully convolutional one-stage 3D object detection on lidar range images. Adv. Neural Inf. Process. Syst. 35, 34899–34911. 10.48550/arXiv.2205.13764

[B39] Wang Y. Wu J. Hovakimyan N. Sun R. (2023). Balanced Training for Sparse Gans. New Orleans.

[B40] Xiao F. Wang H. Li Y. Cao Y. Lv X. Xu G.. (2023). Object detection and recognition techniques based on digital image processing and traditional machine learning for fruit and vegetable harvesting robots: an overview and review. Agronomy 13, 639. 10.3390/agronomy13061625

[B41] Xu X. Duan H. Guo Y. Deng Y. (2020). A cascade adaboost and cnn algorithm for drogue detection in uav autonomous aerial refueling. Neurocomputing 408, 121–134. 10.1016/j.neucom.2019.10.115

[B42] Xu Z. Hrustic E. Vivet D. (2020). “Centernet heatmap propagation for real-time video object detection,” in Computer Vision-ECCV 2020, 16th. European Conference, Glasgow, UK, August 23-28, 2020, Proceedings, Part XXV 16 (Cham: Springer), 220–234. 10.1007/978-3-030-58595-2_14

[B43] Zendrikov D. Solinas S. Indiveri G. (2023). Brain-inspired methods for achieving robust computation in heterogeneous mixed-signal neuromorphic processing systems. Neuromorphic Comput. Eng. 3, 034002. 10.1088/2634-4386/ace64c

[B44] Zhang H. Hu X. Li T. Zhang Y. Xu H. Sun Y.. (2022). Mil series of metal organic frameworks (MOFS) as novel adsorbents for heavy metals in water: a review. J. Hazard. Mater. 429, 128271. 10.1016/j.jhazmat.2022.128271 35093745

[B45] Zhang M. Xie K. Zhang Y.-H. Wen C. He J.-B. (2022). Fine segmentation on faces with masks based on a multistep iterative segmentation algorithm. IEEE Access 10, 75742–75753. 10.1109/ACCESS.2022.3192026

[B46] Zheng Y. Qi Y. Tang Z. Tan J. Koel B. E. Podkolzin S. G.. (2022). Spectroscopic observation and structure-insensitivity of hydroxyls on gold. Chem. Commun. 58, 4036–4039. 10.1039/D2CC00283C 35258054

[B47] Zhou H. Jiang F. Lu H. (2023). SSDA-YOLO: semi-supervised domain adaptive yolo for cross-domain object detection. Comput. Vis. Image Underst. 229, 103649. 10.1016/j.cviu.2023.103649

[B48] Zhu B. Geng T. Jiang G. Guan Z. Li Y. Yun X.. (2023). Surrounding object material detection and identification method for robots based on ultrasonic echo signals. Appl. Bionics Biomech. 2023. 10.1155/2023/1998218

